# Insights into cancer severity from biomolecular interaction mechanisms

**DOI:** 10.1038/srep34490

**Published:** 2016-10-04

**Authors:** Francesco Raimondi, Gurdeep Singh, Matthew J. Betts, Gordana Apic, Ranka Vukotic, Pietro Andreone, Lincoln Stein, Robert B. Russell

**Affiliations:** 1 CellNetworks, Bioquant, Im Neuenheimer Feld 267, University of Heidelberg, 69120 Heidelberg, Germany; 2 Biochemie Zentrum Heidelberg, Im Neuenheimer Feld 328, University of Heidelberg, 69120 Heidelberg, Germany; 3 Cambridge Cell Networks, St. John’s Innovation Centre, Cowley Road, Cambridge CB4 0WS, UK; 4 Department of Medical and Surgical Sciences, University of Bologna and Azienda Ospedaliero-Universitaria di Bologna, Policlinico Sant’Orsola Malpighi, 40138 Bologna, Italy; 5 Ontario Institute for Cancer Research, Toronto, ON M5G 0A3, Canada; 6 Department of Molecular Genetics, University of Toronto, Toronto, ON M5S 1A1, Canada

## Abstract

To attain a deeper understanding of diseases like cancer, it is critical to couple genetics with biomolecular mechanisms. High-throughput sequencing has identified thousands of somatic mutations across dozens of cancers, and there is a pressing need to identify the few that are pathologically relevant. Here we use protein structure and interaction data to interrogate nonsynonymous somatic cancer mutations, identifying a set of 213 molecular interfaces (protein-protein, -small molecule or –nucleic acid) most often perturbed in cancer, highlighting several potentially novel cancer genes. Over half of these interfaces involve protein-small-molecule interactions highlighting their overall importance in cancer. We found distinct differences in the predominance of perturbed interfaces between cancers and histological subtypes and presence or absence of certain interfaces appears to correlate with cancer severity.

High-throughput sequencing is transforming the understanding of cancer. Internationally coordinated efforts now routinely identify somatic mutations arising within tumours, improving the understanding of the disease and driving better diagnostic and therapeutic decisions^1^,^2^. As with most genome-wide sequencing efforts, there are difficulties in identifying causative variants among the thousands typically identified in studies involving hundreds of patients. Previous analyses have defined a few hundred commonly mutated genes and spearheaded the discovery of oncogenes, tumour suppressors and driver mutations^3^,^4^,^5^. Many studies have used functional information, such as pathways^6^, interaction networks^7^, protein domains^8^,^9^ or structure information^10^,^11^,^12^ to prioritize variants. In some instances, this information is also used directly to aid the selection of biomarkers^13^ and/or to aid in patient stratification^14^.

Here we build on these previous efforts by using protein interactions and structures to screen the functional impact of nonsynonymous cancer variants. Using a recently developed method to define the effects of mutations on interfaces involving proteins, DNA/RNA and small-molecules^15^, we defined a set of commonly perturbed interfaces in cancer, and show how these both highlight differences between cancers and identify distinct sub-types within specific cancers. Some subtypes show considerable promise for diagnostics, for example, by predicting survival times more robustly than can be achieved with individual genes.

## Results

### The most affected protein interfaces in cancer

**Figure 1:**
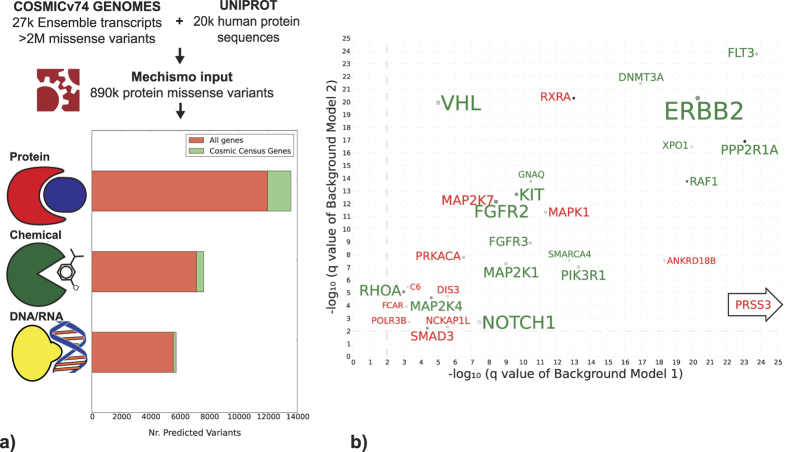
(**a**) Methodological overview: histograms show the counts of predicted protein mutation sites at the interface with protein, chemicals and DNA/RNA. (**b**) Genes mediating the most significantly perturbed interfaces: for each gene, the best q values (FDR) of significantly perturbed, mediated interfaces, is shown. The x-axes and y-axes show q-values obtained from background model 1 and 2. Dots are coloured (grey-black) proportional to the number of unique samples in which a given interface is perturbed, while gene name font size is proportional to the total number of unique samples in which mediated interfaces are significantly perturbed. Dot diameter is proportional to the number of perturbed interfaces. Genes with significantly perturbed interfaces (q <= 0.01 with respect to both background models) in more than 45 unique samples are labelled in green, if they are present in the Cosmic Census or in red if they are not. For space reasons the following significantly perturbed genes (from the Cancer Census) genes are not shown: AKT1, PIK3CA, JAK2, IDH2, IDH1, KRAS, HRAS, PTEN, CHEK2, TP53, GNAS, SMAD4, FBXW7, PPP6C, DICER1, ALK, EGFR, CTNNB1, NRAS, RAC1, SPOP, GNA11. [CHECK THE LIST WITH GURDEEP].

We considered a total of 1,599,218 nonsynonymous, confirmed somatic variant instances from whole genome or exome studies, from a total of 16,535 samples in COSMIC^16^ corresponding to 1,256,900 unique DNA positions, 891,798 unique protein variants and 844,125 protein positions. A total of 304,974 protein changes (34% of total unique protein variants) could be mapped on to at least one three-dimensional (3D)structure using Mechismo^15^. Of these, 26,933 (3%) unique variants were at high-confidence interfaces with other proteins, DNA/RNA or small-molecules (Fig. 1a), representing a total of 10702 unique samples. More than half of these are at interfaces with DNA/RNA or small-molecules (Fig. 1a) which are not considered in studies that focus only on protein-protein interactions^11^.

We used the mapped variants to identify a set of molecular interfaces that are most often perturbed in cancers. For all interfaces, we counted each variant instance across samples from COSMIC (i.e. considering the same variant multiple times if it occurred in multiple samples). We performed the same counts on two shuffled background sets (see Methods) and used these to define 213 highly significant perturbed interfaces (q <= 0.01; see Methods) involving 53 unique mutated genes (Table S1, Fig. 1b). Of these, 95 are protein-protein, 112 are protein-small-molecule and 6 are protein-DNA/RNA interfaces.

Structural knowledge clearly helps pin-point relevant interactions; we repeated the analysis by considering variants on both proteins of each interaction pair (i.e. regardless of whether they were at the interface) and only 100 of 213 interfaces were still significant (see Table S2). An important contribution to the perturbed interface set also comes by considering structures of homologues (see Fig. S1a) as opposed to only structures of the particular human proteins as has been done in other studies^10^. This has important implications for the use of these interfaces. We expect that our set of known protein-protein interface structures is still far from complete^17^, though it is clearly enriched in proteins involved in cancer as these are intensively studied by the structural biology community. However, the tendency for homologous proteins to bind small molecules (e.g. enzymes) or nucleic acids (e.g. transcription factor domains) at similar locations allows the use of representative domain structures for species as far removed from human as yeast or bacteria to create what we expect is a nearly complete set (e.g. ref. 18).

As expected, the majority of interfaces involve proteins that have been previously identified to be altered in cancer. A total of 40 of the 53 (75%) genes are among the 572 gene COSMIC Census^19^ and 35 (13%) are in the 254 gene Cancer 5000 set^4^. For the remaining 11 interface genes that are not in either set, there are varying degrees of evidence in the literature for their involvement in cancer (Table S1a).

These include PRSS3 interacting with calcium ions, ANKRD18B and POLR3B with DNA and NCKAP1L with CYFIPs. By lowering the threshold of unique samples that define significantly perturbed interfaces to 10, the number of significantly perturbed interfaces (407) and affected genes (163) increases, together with the overlap with the Cancer genes census (for a total of 55; Table S1b). Among the remaining candidates, several of have been reported to be linked to cancer in at least one publication, suggesting they could be oncodriver candidates even though they have a lower frequency of variants.

Interestingly, perturbed protein-protein interfaces are almost always unidirectional, in the sense that mutations tend to occur on only one protein (Fig. S1b). For only three (of 95) interfaces the second partner is mutated in more than 20 unique samples (PIK3CA-PIK3R1, EGFR-ERRB2 and SMAD4-SMAD3) and for 38 the second partner had no variants whatsoever.

The predominance of interfaces with small-molecules highlights their importance in cancer. The majority (59 out of 112) involve enzymatic substrates or their analogues, particularly GTP (e.g. in GTPases) and ATP (e.g. in kinases) compounds or their metal ion co-factors (Table S1, Fig. 1), and the majority of the oncogenic mutations in our dataset lie on these sites. The binding interfaces of metal co-factors with structural stabilization roles are also often perturbed by cancer missense variants (Table S1, Fig. 1). The most prominent example is the TP53/zinc ion interface, which appears to be informative about cancer severity (see below).

### Cancer types show differences in perturbed interface profiles

**Figure 2:**
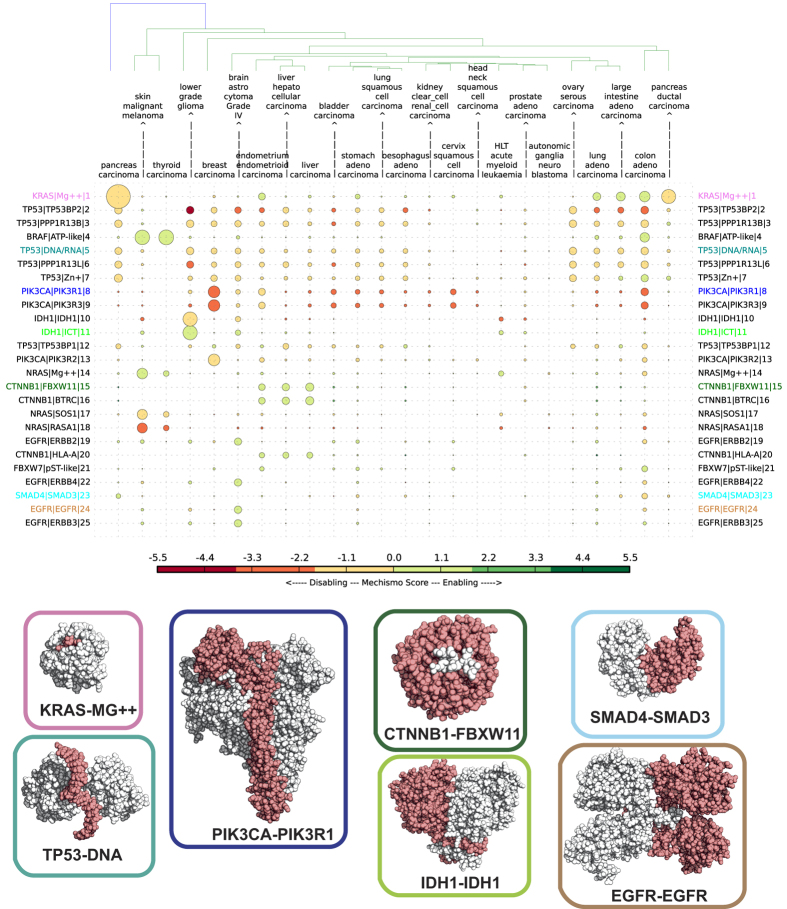
Cancer type specific interface perturbation matrix: for each of the most abundant 24 cancer types (columns), the 25 most frequently perturbed interfaces are shown (rows). Each dot represents a perturbed interface, with the diameter being proportional to the sample frequency and the colour corresponding to the median of the Mechismo scores. When the same perturbed protein interface binds multiple chemicals (e.g.for KRAS or NRAS), only the one perturbed in the highest number of unique samples is reported. Space-filling representations of representative most perturbed interfaces (with the protein most affected by mutations coloured in white and the interacting partner in red): KRAS-MG++ (PDB ID: 5P21), TP53-DNA (PDB ID: 1TUP), PIK3CA-PIK3R1 (PDB ID: 3HMM), IDH1-IDH1 (PDB ID: 1T09), CTNNB1-FXBW11 (PDB ID: 1P22), EGFR-EGFR (PDB ID: 1IVO), SMAD4-SMAD3 (PDB ID: 1U7F).

We constructed a matrix of how these perturbed interfaces are affected in each of the most common cancer types (Fig. 2, Fig. S2). In doing so, we also considered whether or not each variant had a predicted enabling or disabling effect on the interface^15^. Broadly, the Mechismo approach uses residue interaction statistical pair potentials (based on frequencies in known interfaces) to assess the effect of changing any interface residue to another (Methods). This process uncovers mechanistic insights into individual cancers. For example, mutations in malignant melanoma are predicted to disable the RAC1-DOCK1 interface but enable interactions between RAC1 and PAK3 (Figs S2 and S3). We also see many known differences between cancers that were discovered when considering mutated genes alone. For instance, those affecting the same tissue with distinct histologies, such as lung squamous cell carcinoma compared to adenocarcinoma (Fig. 2 and Fig. S2), the latter mainly differing for mutations of KRAS and EGFR^20^,^21^. We also identify known cancer specific mutations in PIK3CA, BRAF, IDH1 and other genes and as expected, TP53 perturbations are found in most cancers^3^,^4^.

**Figure 3:**
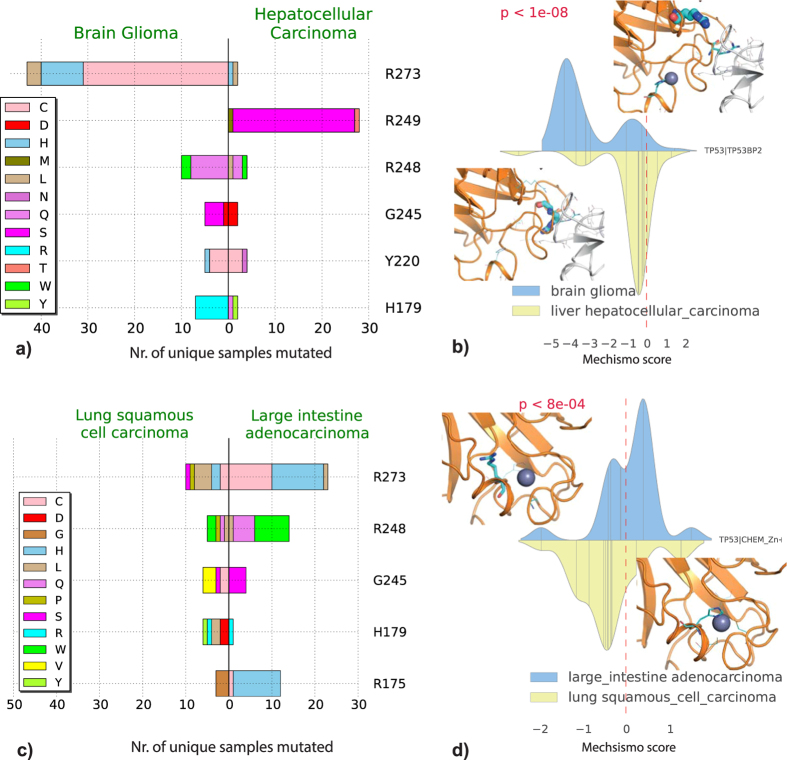
TP53 interfaces significantly different in cancers: (**a**) mutational spectra of TP53 mutations in Glioma and Hepatocellular carcinoma (count cutoff ≥5) perturbing the interface with TP53BP2. (**b**) Mechismo score distribution and structure captions for the TP53 and TP53BP2 interface in Glioma and Hepatocellular carcinoma. Mutated residues are shown as sticks whose radius is proportional to the mutation count. Representations in (**c**,**d**) are the same as in (**a**,**b**), respectively, for the TP53-ZN^++^ interface in Lung Squamous Cell Carcinoma and Large Intestine Adenocarcinoma. In the density plots of Mechismo score distributions, bar x coordinates indicate variant Mechismo scores and the height is proportional to the number of samples containing variants.

However, several differences are only apparent when considering perturbed interfaces. The most striking data are related to the tumour suppressor TP53, for which cancers often show distinct mutational preferences. Of 105 interfaces where perturbations differ significantly between cancers (p <= 0.05), 60 involve TP53. Perturbations vary with regard to the co-regulators TP53BP1/2, DNA and zinc ions (Fig. 2). Certain cancers show different effects at particular interfaces, such as TP53-TP53BP2, which is predicted to be strongly disabled in low grade glioma and weakly disabled in hepatocellular carcinoma owing to different mutational preferences of the principal contacting residues (Fig. 3a,b). Similarly, the TP53 zinc ion interface is disabled in lung squamous cell carcinoma and enabled in large intestine adenocarcinoma (Fig. 3c,d).

A similar situation is seen in β-catenin-1 (CTNNB1). In four cancers, mutations at the N-terminal disordered region of the protein are predicted to perturb interactions with BTRC, FBXW11 and HLA-A, whereas only two of these cancers (liver cancers) have additional mutations at the armadillo region perturbing interactions with other proteins (Fig. S5). These are consistent with a weaker activation of the β-catenin pathway compared to mutations occurring at the N-terminal region^22^.

Particular interface perturbations involving specific amino acid changes in GTPases appear to be differentially selected among different cancer types and subtypes, potentially modulating interfaces differently as suggested previously for RHOA^23^. For example, in malignant melanoma the most common oncogenic mutations at Gln-61 in NRAS (Q61R,K) are predicted to disable the guanine exchange factor SOS1 (as previously reported^24^) and activator RASA1 interactions, whereas in pancreatic carcinoma KRAS mutations are more enabling of both of these proteins (Fig. S6b,c). Mutations at the equivalent residue (Q209P,L) on both GNAQ and GNA11 α-subunits of heterotrimeric G proteins are predicted to perturb the interaction with regulatory β-subunits and Regulator of G protein signalling (RGS), as well as with the effector phospholipase C beta 3 (PLCB3) (Table S1, Fig. S6d). This suggests that the oncogenic effect of mutations of this conserved glutamine might result from modulation of interactions with regulatory/effector proteins, in addition to the effects on intrinsic and GAP-mediated GTP hydrolysis^25^. In contrast, highly oncogenic mutations of Arg-201 on GNAS, which also interfere with nucleotide hydrolysis^26^, are not predicted to significantly perturb any protein interface. KRAS/NRAS Q61 and GNAQ/11 Q209 codons do not explain the observed mutational preferences among different variants. We speculate that these derive from different requirements to interact with effectors/regulators, as the predictions suggest and *in vitro* characterizations have begun to uncover^24^,^27^.

### Mechanistic differences define clinical sub-populations and can help predict outcome

**Figure 4:**
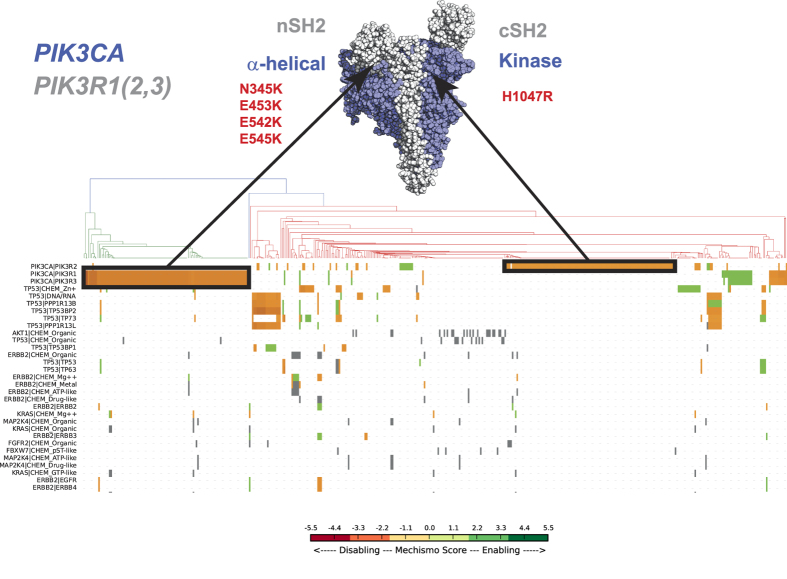
Breast carcinoma clustering based on Mechismo predictions: each column of the clustering matrix represent a sample, while each perturbed interface is represented on each row. Each matrix element is coloured according to Mechismo score, ranging from red (disabling) to green (enabling). Mutually exclusive set of mutations of PIK3CA predicted to perturb the interaction with PIK3CA regulatory proteins are highlighted in blue on the clustering matrix as well as on a space-filling representation of PIK3CA and PIK3R1 and 2. The structure shows the C-terminal SH2 domain (cSH2) of PIK3R2 from a complex with PIK3CA (PDB: 2Y3A) previously fitted to a complex between PIK3CA-PIK3R1 (PDB: 3HMM).

Sub-classification of patients within the same cancer type can have critically important consequences, for instance in selecting the most effective therapy. We tested whether the mechanistic differences within perturbed interfaces could uncover known sub-populations by clustering samples according to their perturbation profiles (Fig. 4 and Fig. S7). This process identifies clear examples of distinct and mutually exclusive mechanisms within different cancer types, described below. For these sub-populations, we also tested whether they showed any evidence of specific clinical phenotypes as measured by donor information (vital & disease status and survival time) from the ICGC data portal^2^ and whether the classification would be evident when considering the mutated genes alone (i.e. without structural information).

We started by looking at perturbed interfaces leading to characteristic sub-populations patterns in specific cancer types. Different mutations on Phosphoinositide-3-kinase α catalytic subunit (PIK3CA) correlated with two distinct (p = 1.97e^−12^, q = 2.36e^−10^; Table S3a) breast cancer subtypes: one defined by mutations in the α−helical domain that are predicted to disable interfaces with regulator N-terminal SH2 domains, and another that affects the highly oncogenic kinase domain mutation (H1047R) and is predicted to disable the C-terminal SH2 domain interface (Fig. 4). Interestingly, the N-terminal SH2 domain perturbed group is mutually exclusive with mutations affecting the interfaces of TP53 with DNA or co-regulators (p = 1.23e^−3^, q = 4.47e^−2^; Table S3a), with the latter being associated with poorer prognosis in breast carcinoma (a lower likelihood to completely remit: p = 6.17e^−3^, q = 8.63e^−2^; see Table S3a). On the other hand, samples bearing PIK3CA mutations perturbing the N-terminal SH2 domain of regulatory subunits are associated with poorer prognosis in low grade glioma (p = 1.4e^−3^, q = 3.79e^−2^; Table S3a).

ALK mutations in autonomic ganglia neuroblastoma are principally located in two distinct kinase domain regions (Phe-core and αC/A-loop)^14^ and perturb different interfaces in a mutually exclusive fashion (p = 1.06e^−2^, q = 3.18e^−2^; Table S3a). In line with a recent report of the therapeutic stratification potential of neuroblastoma patients based on ALK genomic status^14^, samples with mutations of the second group (including R1275), which are predicted to perturb a phosphosite-mediated dimerization interface in addition to the ATP binding pocket, are characterized by a different distribution of survival times in autonomic ganglia neuroblastoma (ranksum, p = 3.4e^−2^; Table S3a) and a significantly increased risk across cancers (Cox, p = 2.8e^−3^; Table S5b). Similarly, mutations perturbing the ATP binding pocket of the RPS6KA3 kinase are associated to a significant increased risk in liver cancers (Cox, p = 8.65e^−4^; Table S5a) as well as cross-cancer (p = 3.71e^−2^; Table S5b,c). The severity of these mutations is in line with the RPS6KA3 suppressive role of the RAS-MAPK pathway which is lost upon inactivating mutation^28^.

Strong mutual exclusivity of certain perturbed interfaces is still observed when considering all cancers together (Fig. S7), where subtypes defined on the basis of this larger set also reveal distinct properties that link to cancer severity. Indeed, sample clusters with a predominance of KRAS-GTP-like perturbations (or IDH1-Mg^++^/IDH1-IDH1, or TP53 dimers) have a poorer prognosis in terms of complete remission compared to sample groups with either PIK3CA mutations perturbing PIK3R1 or PIK3R2 (Table S4e).

**Figure 5:**
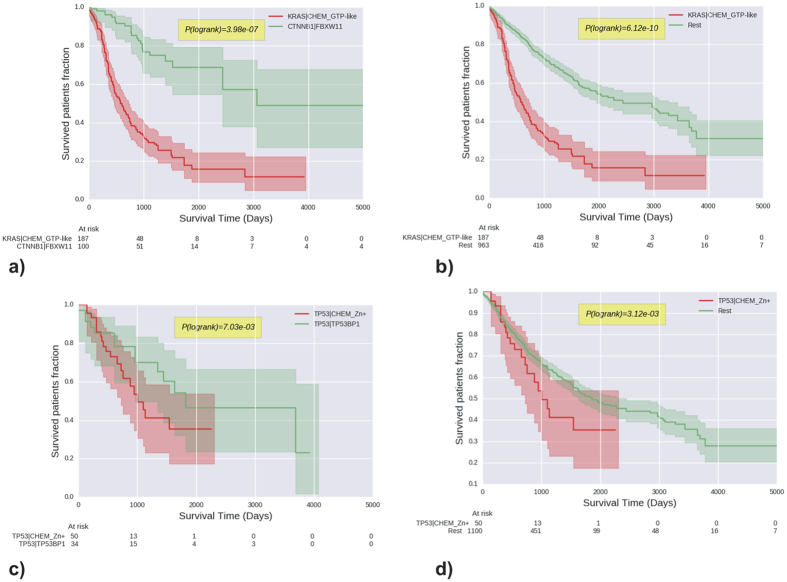
Survival time analysis: Kaplan-Meier survival analysis plots and logrank test probabilities for patient samples with mutually exclusive mutations affecting respectively KRAS-GTP/CTNNB1-FBXW11 and TP53-TP53BP1/TP53-Zn^++^ interface pairs (**a**,**c**) and of KRAS-GTP and TP53-Zn^++^ interfaces only (**b**,**d**).

Mutations affecting the KRAS/GTP interface and those targeting the CTNNB1 interfaces with FBXW11 or BTCR are largely mutually exclusive when considering all cancer types (Fig. S7; Table S3c), and particularly for endometrial adenocarcinoma, where both mutations are common. KRAS/GTPase mutations have a shorter survival and higher hazard ratio (Cox, p = 3.67e^−27^; Table S5) than those within CTNNB1 (Fig. 5a), though this is also apparent when considering genes alone (i.e. without the interface context; Fig. S9).

In some instances, different mutations of the same genes perturbing alternative interaction interfaces are associated to distinct clinical features of affected individuals. For example, PIK3CA mutations predicted to disable interfaces with regulator N-terminal SH2 domains (above) have a statistical significant cross-cancer lower survival and higher hazard ratio (Cox, p = 1.3e^−2^; Table S5b,c) than those predicted to affect the C-terminal SH2 domains.

Different interfaces involving TP53 define distinct sub-populations within several cancer types, such as rectum adenocarcinoma, esophageal adenocarcinoma, ovary serous adenocarcinoma, pancreatic adenocarcinoma and low grade glioma (Table S3a, Fig. S8), with one group being defined by mutations affecting DNA/regulator binding and another defined by those affecting zinc ion binding (and thus most likely leading to an unfolded, non-functional protein^29^).These appear to be distinct evolutionary trajectories where TP53 function is altered either towards specific interactions or knocked-out altogether.

Interestingly, when considering all cancers, there is a significant difference in overall survival among these TP53 interface-defined tumour subtypes, with variants at the zinc binding site being associated with poorer overall survival (logrank, p = 3,12e^−3^; Fig. 5d) as well as compared to mutually exclusive TP53 mutations perturbing alternative interfaces (i.e. TP53BP1; Fig. 5c, Table S2c). Consistently, individuals affected by the latter variants have the greatest estimated hazard ratio compared to the other TP53-mediated interfaces (Table S5b) across all cancer types. Inspection suggests that this observation is mostly the result of liver cancers & pancreatic carcinoma patients in the Zinc perturbed group (Tables S3d and S5b).

## Discussion

The full value of HTS data is only apparent when considering genetic variants beside information about biological mechanism. We have shown that considering mechanism, in the form of perturbed interfaces, reveals insights that are not apparent when considering genes in isolation. Naturally, our findings are restricted to missense mutations and are not necessarily applicable to oncogenic mutations that, for instance, lead to increased expression or copy number.

In a recent pan-cancer survey of missense variants in protein structures^10^, protein-mediated interfaces with either proteins, chemicals or DNA/RNA have also been considered in the context of 3D clustering. Additionally, a recent structure-based overview of missense cancer variants affecting protein-protein interactions has been reported^11^, focusing on the identification of those interfaces significantly enriched in non-synonymous mutations. Here we have quantitatively predicted the functional consequences of substitutions (i.e. enabling or disabling effects) at protein interfaces in a cancer type-specific fashion. Moreover, variants at multiple, not necessarily spatially adjacent, sites might contribute to perturb the same interface with a specific interactor, allowing the identification of new candidate genes not previously reported. We have also related differences across interfaces more comprehensively to cancer phenotypes suggesting that ultimately they can be used diagnostically.

Although our method also considers structural information from homologous proteins, our findings are still limited by the availability of structural information for the proteins considered. For cancer, the community profits from a vast number of cancer related structures, though for other diseases this is not the case^30^, and indeed even for cancer there are many major players with limited structural characterization (e.g. BRCA2). Fortunately, the pace of structure determination, particularly for protein interactions and complexes, has increased rapidly in the last decade, meaning that an ever greater set of structures will be available for investigations like this one.

Ultimately one would also wish to use subtypes to aid therapeutic decisions, though currently this is hampered by the relative paucity of data on the particular treatments and outcomes for publically available samples. The eventual availability of wider datasets will thus likely allow studies like this to impact ultimately on cancer therapy and patient well-being.

## Materials and Methods

### Cancer mutation data

We extracted confirmed somatic, missense, nonsynonymous mutations from version 74 of COSMIC genomes (http://cancer.sanger.ac.uk/wgs). We mapped 22896 of 27547 (83%) of the associated Ensembl transcripts to Uniprot canonical (Swissprot) isoforms, which left 891,798 unique protein mutations of which 304,974 could be mapped to one or more 3D structures.

### Defining perturbed interfaces

We predicted functional consequences of COSMIC missense mutations using Mechismo^15^ (mechismo.russelllab.org), which matches protein sequence position to positions within structures and identifies sites affecting interactions with other proteins, DNA/RNA or small-molecules. We considered high confidence predictions for protein-protein interactions, which includes known structures or close (>=70% sequence identity) homologs and only very confident, physical protein-protein interactions (as defined by Mechismo based a benchmark for the accuracy of perturbed interfaces)^15^. For chemical and DNA/RNA we also considered predictions with low/medium confidence (as low as 30% sequence identity). The lower thresholds for chemicals and DNA/RNA were based on the observation that these binding sites are generally correctly predicted a low sequence identities, even when the precise details of the contacts are not (e.g. as defined in the original Mechismo paper)^15^.

We identified the most perturbed interactions in cancer by ranking each interacting pair based on the number of unique samples where a missense mutation was predicted to affect the interface. We tested the significance of the most perturbed interactions in COSMIC by using two different interactome perturbation random models.

We defined two background models. For the first (BM1), we randomly shuffled the observed substitutions among positions with the same amino acid in the same protein and, in the second (BM2), we considered any position in the same protein (regardless of amino acid) and chose a random amino acid change. We obtained Mechismo data for both background sets as for the original data (above).

We then calculated the probability of getting the same number of observed perturbing events for each interaction by chance, through a binomial test


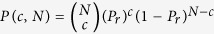


where *N* is the total number of samples, *c* is the number of unique samples in which a given interaction has been found perturbed and *P*_*r*_ is the probability, from the background random distribution, to get the same interface perturbed. The obtained values were corrected through the False Discovery Rate (FDR)/Benjamini-Hochberg procedure (to give q-values). Interfaces having a q-value below 0.01 and that are perturbed in at least 20 unique samples were considered significant.

To build the interface perturbation matrix in Figs 2 and S2, we considered the same variant multiple times if it occurred in multiple samples. In case of multiple variants affecting the same interaction interface within the same sample, we combined the Mechismo interactions scores of the involved sites. This led to an overall perturbation effect for all the considered protein-protein, protein-chemical and protein-DNA/RNA interacting pairs in each sample.

To estimate the overall perturbation effect of each interacting pair in a particular cancer or sub-type, we calculated the median of the distribution of the Mechismo score. This information was assembled into cancer type-specific fingerprints that we used to cluster all types based on their similarity. From each of the top 30 cancer types (based on number of samples), we considered the top 30 most perturbed gene pairs. We further retained only those interfaces perturbed in at least 20 samples of at least one of the top cancer types, and that were significant in both background models, obtaining a final list of 48 gene pairs in 24 cancer types (Fig. S2).

We defined cancer types using the COSMIC classification system considering Primary tissue/Tissue sub-type1 and Primary histology/Histology sub-type1 specifications.

### Clustering and mutual exclusivity analysis

We clustered samples by hierarchical, complete linkage clustering of the Mechismo interaction scores (above), and defined clusters using a depth cutoff of 0.9 (deduced by visual inspection of the data). We evaluated mutual exclusivity of interaction perturbations in each cancer type as well as in all cancers together for interacting pairs found perturbed in at least 10 and 20 unique samples respectively. We defined significantly mutually exclusive interface pairs as those with a one-tailed, Fisher exact test P-value smaller than 0.1 after FDR correction.

### Relating perturbed interfaces to survival time

We collected donor information (10,805 donors) from ICGC (icgc.org), release 19, and matched these to the corresponding COSMIC sample. We considered vital status (alive/deceased), disease status (complete remission or not – i.e. partial remission, relapse, progression) and survival time, leading to 1150 unique samples with complete clinical information and available mechismo predictions.

We assessed statistical significance of the association of clusters (above) with vital or disease status through a two-tailed Fisher exact test. To check for significant differences between groups in terms of survival time we compared the distribution of survival time through a Mann-Witney U-test. Kaplan-Meier survival analysis plots were generated for groups significantly differing for their survival time distributions and the statistical significance of survival curve’s differences was evaluated through a logrank test. Cox’s proportional hazard model were employed to predict hazard ratios and survival probability of patients affected by interface-perturbing mutations, employing age, sex and cancer type as covariates.

All the clustering and statistical analysis have been done in python (www.python.org/) through scipy (www.scipy.org/), statsmodels (statsmodels.sourceforge.net/) and lifelines (lifelines.readthedocs.org/en/latest/) libraries.

## Additional Information

**How to cite this article**: Raimondi, F. *et al*. Insights into cancer severity from biomolecular interaction mechanisms. *Sci. Rep*. **6**, 34490; doi: 10.1038/srep34490 (2016).

## Supplementary Material

Supplementary Information

Supplementary Table 1a

Supplementary Table S1

Supplementary Table S2

Supplementary Table S3

Supplementary Table S4
