# Corrigendum: Insights into cancer severity from biomolecular interaction mechanisms

**DOI:** 10.1038/srep46284

**Published:** 2017-05-03

**Authors:** Francesco Raimondi, Gurdeep Singh, Matthew J. Betts, Gordana Apic, Ranka Vukotic, Pietro Andreone, Lincoln Stein, Robert B. Russell

Scientific Reports 6: Article number: 34490; 10.1038/srep34490 published online: 10 04 2016; updated: 05 03 2017.

This Article contains a typographical error. In the Results section, under the heading ‘The most affected protein interfaces in cancer’,

‘EGFR-ERRB2’

should read:

‘EGFR-ERBB2’.

