# Supplementary material for: Insights into cancer severity from biomolecular interaction mechanisms
Source: Sci Rep. 2016 Oct 4;6:34490. doi: 10.1038/srep34490 (PMC5048291; doi:10.1038/srep34490)
Supplement: Supplementary Information [file srep34490-s1.pdf]

SUPPLEMENTAL INFORMATION

Insights into cancer severity from biomolecular interaction mechanisms

Francesco Raimondi[§](1,2), Gurdeep Singh[§](1,2), Matthew J. Betts(1,2), Gordana Apic(1,3), Ranka Vukotic(4), Pietro Andreone(4), Lincoln Stein(5,6) and Robert B. Russell(1,2)*

1. CellNetworks, Bioquant, Im Neuenheimer Feld 267
2. Biochemie Zentrum Heidelberg, Im Neuenheimer Feld 328
University of Heidelberg
69120 Heidelberg, Germany
3. Cambridge Cell Networks, St. John’s Innovation Centre, Cowley Road, Cambridge CB4 0WS, UK
4. Department of Medical and Surgical Sciences, University of Bologna and Azienda Ospedaliero-Universitaria di Bologna, Policlinico Sant’Orsola Malpighi, 40138 Bologna, Italy
5. Ontario Institute for Cancer Research, Toronto, ON M5G 0A3, Canada
6. Department of Molecular Genetics, University of Toronto, Toronto, ON M5S 1A1, Canada

*Corresponding author: Tel: +49 6221 54 51 362
Email: robert.russell@bioquant.uni-heidelberg.de
[§]Equal contributions

Supplemental figure legends

Figure S1

a) Degree of homology for the templates used for the predictions.

b) Most perturbed interfaces directionality

Figure S2

Extended cancer type specific interface perturbation matrix: for each of the most abundant 24 cancer types (columns), the 48 most frequently perturbed interfaces are shown (rows). Each dot represents perturbed interfaces, with the diameter being proportional to the sample frequency and the colour corresponding to the median of the mechismo scores.

Figure S3

Mechismo score distribution of most significantly diverging interfaces mediated by the same gene in a given cancer type.

Figure S4

Mechismo score distribution of interfaces most significantly diverging between different cancer types.

Figures S5

Mutational spectra of the CTNNB1 gene in four different cancer types. Interactors whose interfaces are perturbed are listed in green at the bottom of the plot.

Figures S6

a) Mutational spectra of NRAS and KRAS in skin malignant melanoma and pancreas carcinoma. **b)** Structure caption and mechismo score distributions of variant predicted effects towards K and NRAS interfaces with SOS1 (top) and RASA1 (bottom). **c)** Predicted effect for Q61R mutation to H,K in NRAS. Red and green lines indicate disabling and enabling effects respectively. Solid, dashed and dotted lines indicate the level of confidence of the prediction (respectively high, medium and low) while the line thickness is proportional to

the score magnitude. **d)** Same representation as in c) for predicted effects of mutations Q209 P, L of GNAQ and GNA11

Figures S7

Matrix representation of the clustering obtained by considering samples from all cancer types.

Figures S8

Clustering matrix of four major cancer types showing the mutual exclusivity between TP53 interfaces mediated with ZN++ compared toDNA /co-regulation of transcription proteins.

Figures S9

Comparison of Kaplan-Meier survival plots for KRAS and CTNNB1 perturbed interfaces (green and red) and genes only (lime and orange).

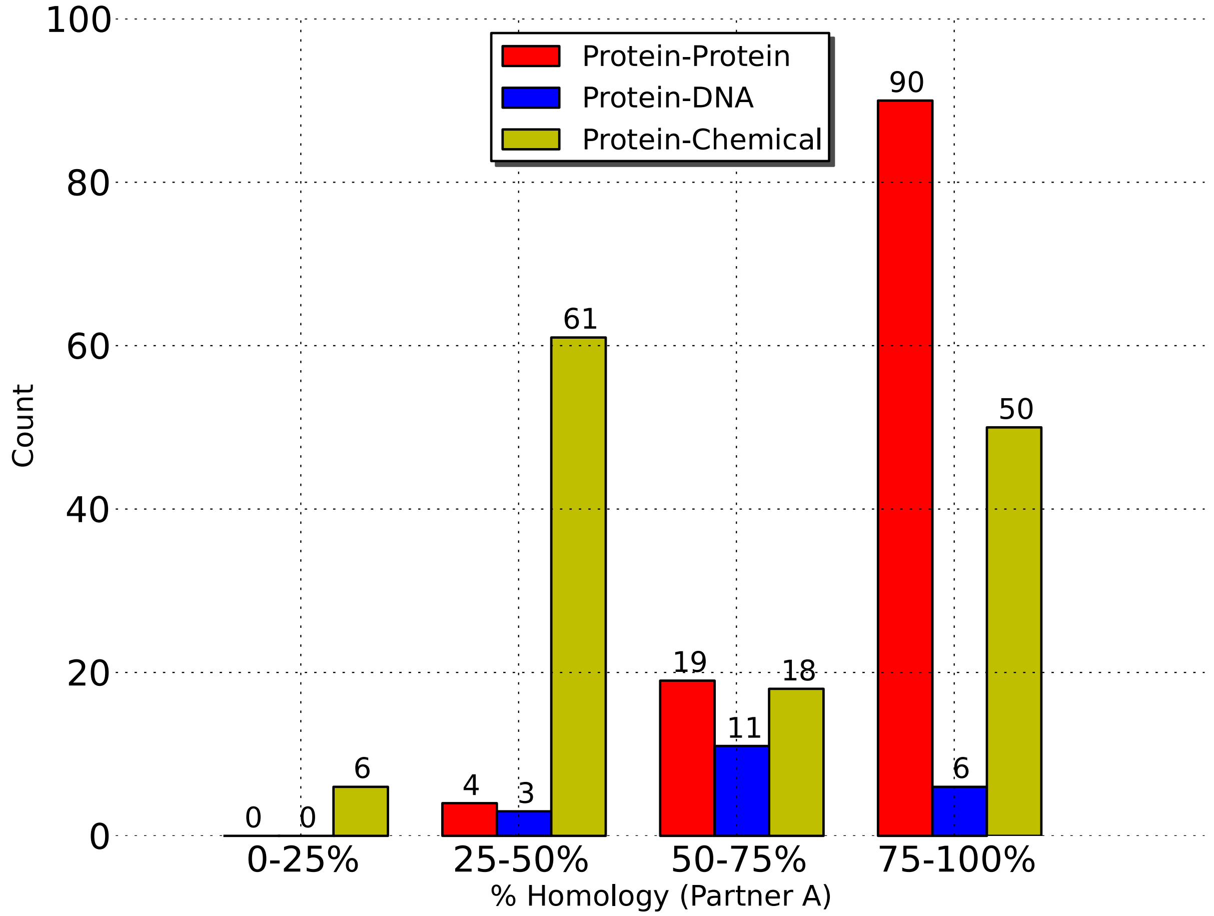
Protein-Protein
Protein-DNA
Protein-Chemical
Count
0
20
40
60
80
100
0 0 6
4 3 61
19 11 18
90 6 50
0-25%
25-50%
50-75%
75-100%
% Homology (Partner A)

a)

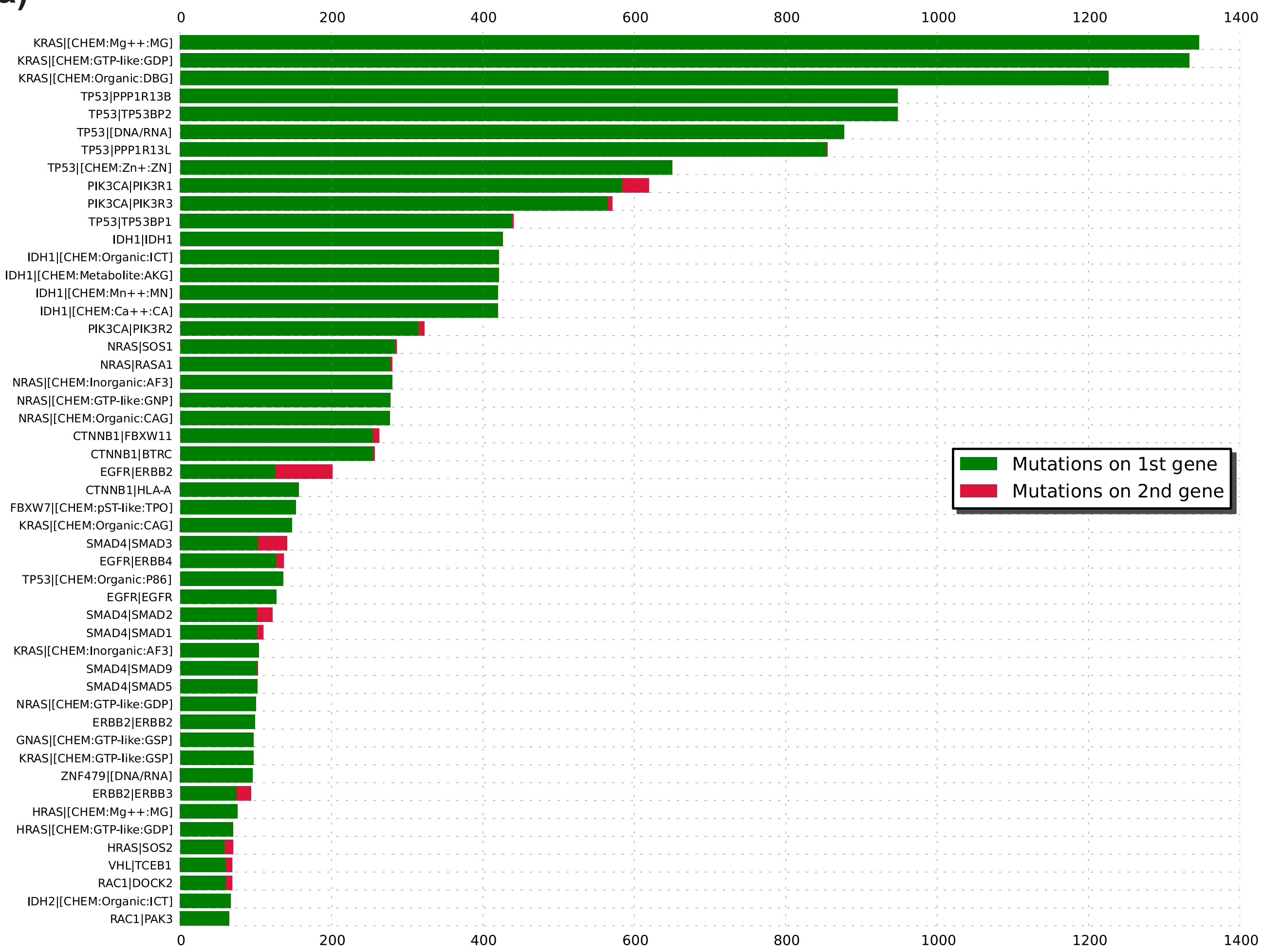
0
200
400
600
800
1000
1200
1400
KRAS|[CHEM:Mg++:MG]
KRAS|[CHEM:GTP-like:GDP]
KRAS|[CHEM:Organic:DBG]
TP53|PPP1R13B
TP53|TP53BP2
TP53|[DNA/RNA]
TP53|PPP1R13L
TP53|[CHEM:Zn+:ZN]
PIK3CA|PIK3R1
PIK3CA|PIK3R3
TP53|TP53BP1
IDH1|IDH1
IDH1|[CHEM:Organic:ICT]
IDH1|[CHEM:Metabolite:AKG]
IDH1|[CHEM:Mn++:MN]
IDH1|[CHEM:Ca++:CA]
PIK3CA|PIK3R2
NRAS|SOS1
NRAS|RASA1
NRAS|[CHEM:Inorganic:AF3]
NRAS|[CHEM:GTP-like:GNP]
NRAS|[CHEM:Organic:CAG]
CTNNB1|FBXW11
CTNNB1|BTRC
EGFR|ERBB2
CTNNB1|HLA-A
FBXW7|[CHEM:pST-like:TPO]
KRAS|[CHEM:Organic:CAG]
SMAD4|SMAD3
EGFR|ERBB4
TP53|[CHEM:Organic:P86]
EGFR|EGFR
SMAD4|SMAD2
SMAD4|SMAD1
KRAS|[CHEM:Inorganic:AF3]
SMAD4|SMAD9
SMAD4|SMAD5
NRAS|[CHEM:GTP-like:GDP]
ERBB2|ERBB2
GNAS|[CHEM:GTP-like:GSP]
KRAS|[CHEM:GTP-like:GSP]
ZNF479|[DNA/RNA]
ERBB2|ERBB3
HRAS|[CHEM:Mg++:MG]
HRAS|[CHEM:GTP-like:GDP]
HRAS|SOS2
VHL|TCEB1
RAC1|DOCK2
IDH2|[CHEM:Organic:ICT]
RAC1|PAK3
Mutations on 1st gene
Mutations on 2nd gene

b)

Figure S1

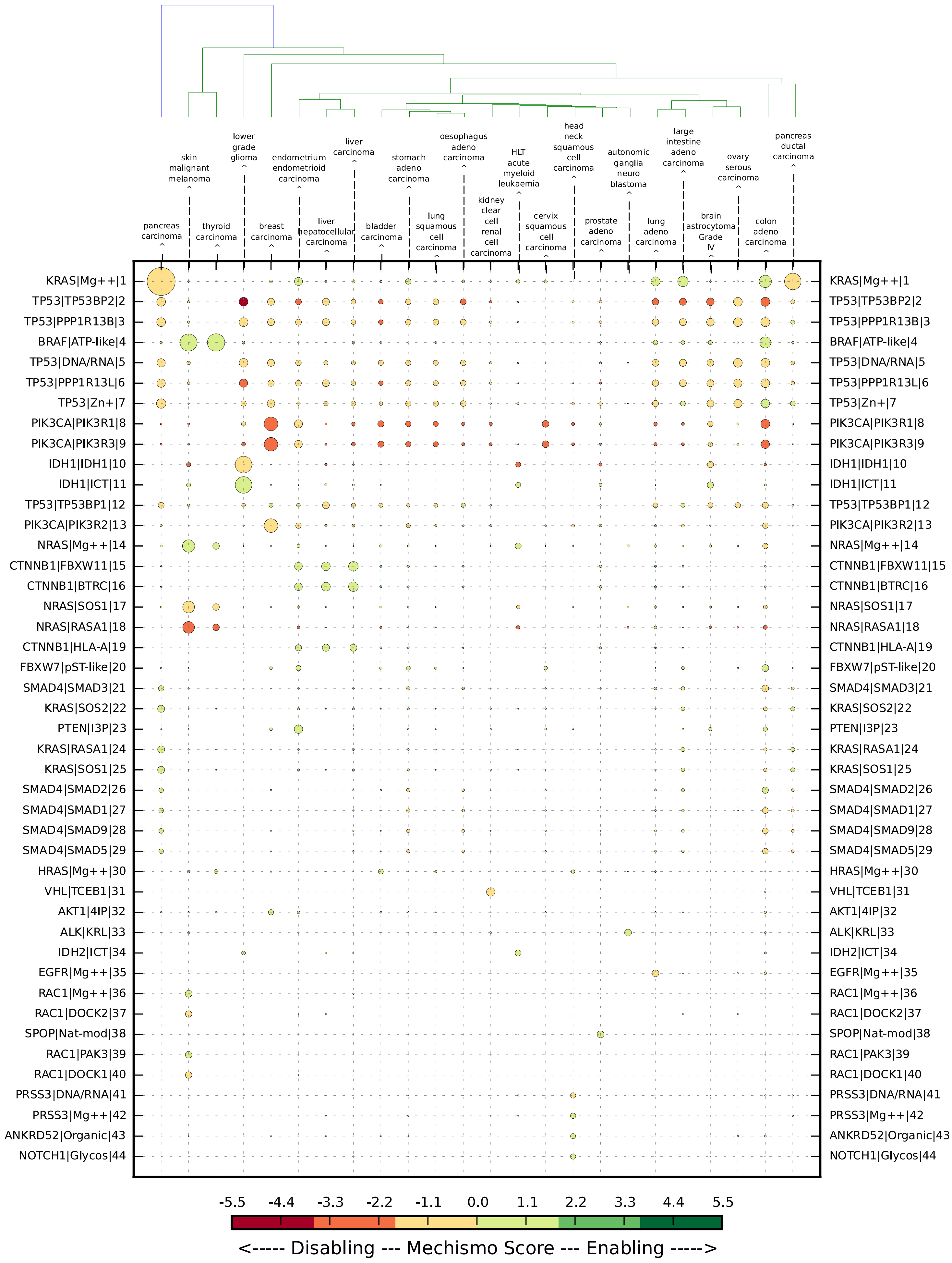

pancreas carcinoma
skin malignant melanoma
thyroid carcinoma
lower grade glioma
breast carcinoma
endometrium endometrioid carcinoma
liver hepatocellular carcinoma
liver carcinoma
bladder carcinoma
stomach adeno carcinoma
lung squamous cell carcinoma
oesophagus adeno carcinoma
kidney clear cell renal cell carcinoma
HLT acute myeloid leukaemia
cervix squamous cell carcinoma
head neck squamous cell carcinoma
prostate adeno carcinoma
autonomic ganglia neuro blastoma
lung adeno carcinoma
large intestine adeno carcinoma
brain astrocytoma Grade IV
ovary serous carcinoma
colon adeno carcinoma
pancreas ductal carcinoma
KRAS|Mg++|1
TP53|TP53BP2|2
TP53|PPP1R13B|3
BRAF|ATP-like|4
TP53|DNA/RNA|5
TP53|PPP1R13L|6
TP53|Zn+|7
PIK3CA|PIK3R1|8
PIK3CA|PIK3R3|9
IDH1|IDH1|10
IDH1|ICT|11
TP53|TP53BP1|12
PIK3CA|PIK3R2|13
NRAS|Mg++|14
CTNNB1|FBXW11|15
CTNNB1|BTRC|16
NRAS|SOS1|17
NRAS|RASA1|18
CTNNB1|HLA-A|19
FBXW7|pST-like|20
SMAD4|SMAD3|21
KRAS|SOS2|22
PTEN|I3P|23
KRAS|RASA1|24
KRAS|SOS1|25
SMAD4|SMAD2|26
SMAD4|SMAD1|27
SMAD4|SMAD9|28
SMAD4|SMAD5|29
HRAS|Mg++|30
VHL|TCEB1|31
AKT1|4IP|32
ALK|KRL|33
IDH2|ICT|34
EGFR|Mg++|35
RAC1|Mg++|36
RAC1|DOCK2|37
SPOP|Nat-mod|38
RAC1|PAK3|39
RAC1|DOCK1|40
PRSS3|DNA/RNA|41
PRSS3|Mg++|42
ANKRD52|Organic|43
NOTCH1|Glycos|44
-5.5 -4.4 -3.3 -2.2 -1.1 0.0 1.1 2.2 3.3 4.4 5.5
<----- Disabling --- Mechismo Score --- Enabling ----->

Figure S2

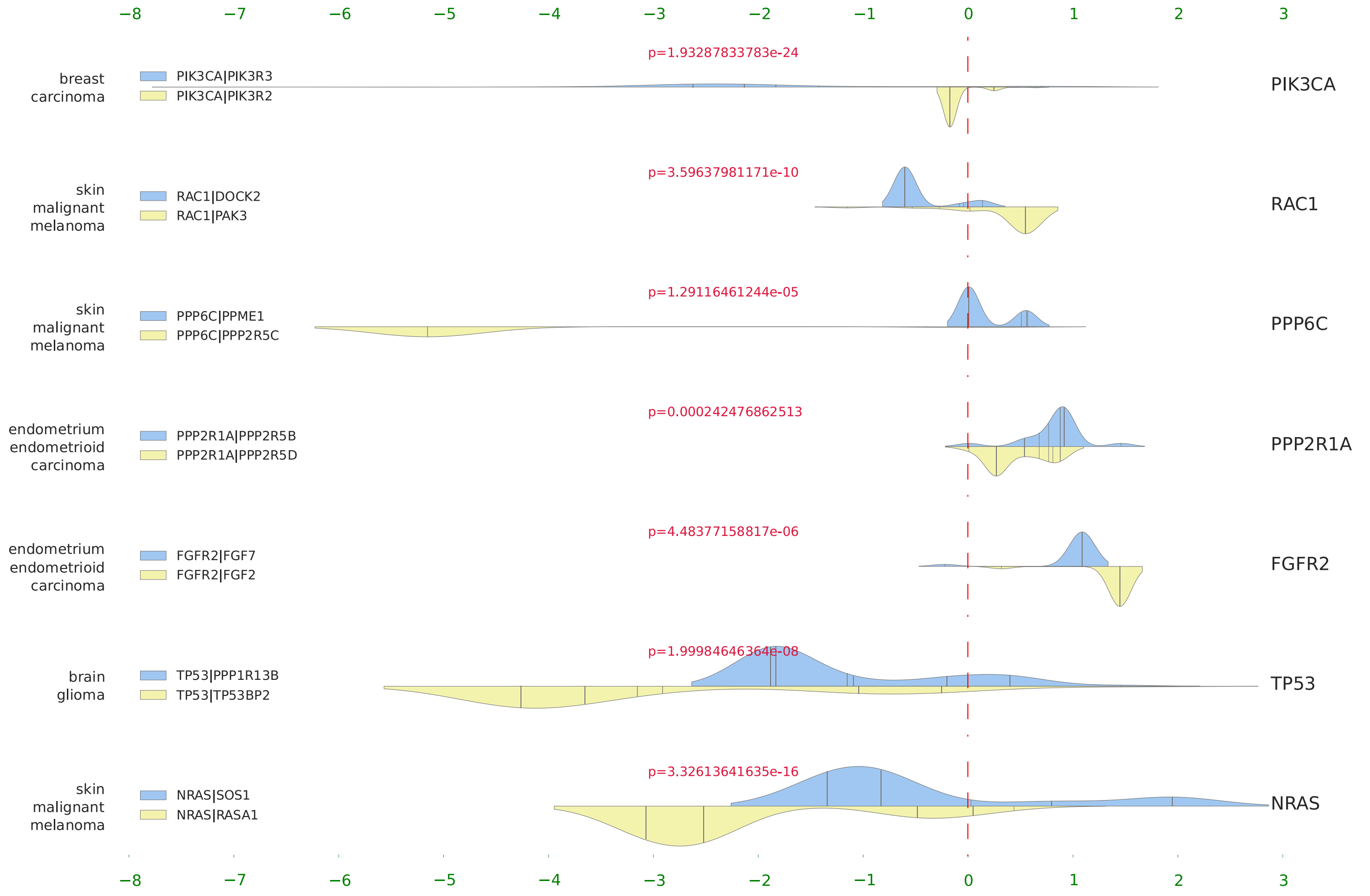
−8 −7 −6 −5 −4 −3 −2 −1 0 1 2 3
breast carcinoma
PIK3CA|PIK3R3
PIK3CA|PIK3R2
p=1.93287833783e-24
PIK3CA
skin malignant melanoma
RAC1|DOCK2
RAC1|PAK3
p=3.59637981171e-10
RAC1
skin malignant melanoma
PPP6C|PPME1
PPP6C|PPP2R5C
p=1.29116461244e-05
PPP6C
endometrium endometrioid carcinoma
PPP2R1A|PPP2R5B
PPP2R1A|PPP2R5D
p=0.000242476862513
PPP2R1A
endometrium endometrioid carcinoma
FGFR2|FGF7
FGFR2|FGF2
p=4.48377158817e-06
FGFR2
brain glioma
TP53|PPP1R13B
TP53|TP53BP2
p=1.99984646364e-08
TP53
skin malignant melanoma
NRAS|SOS1
NRAS|RASA1
p=3.32613641635e-16
NRAS
−8 −7 −6 −5 −4 −3 −2 −1 0 1 2 3

Figure S3

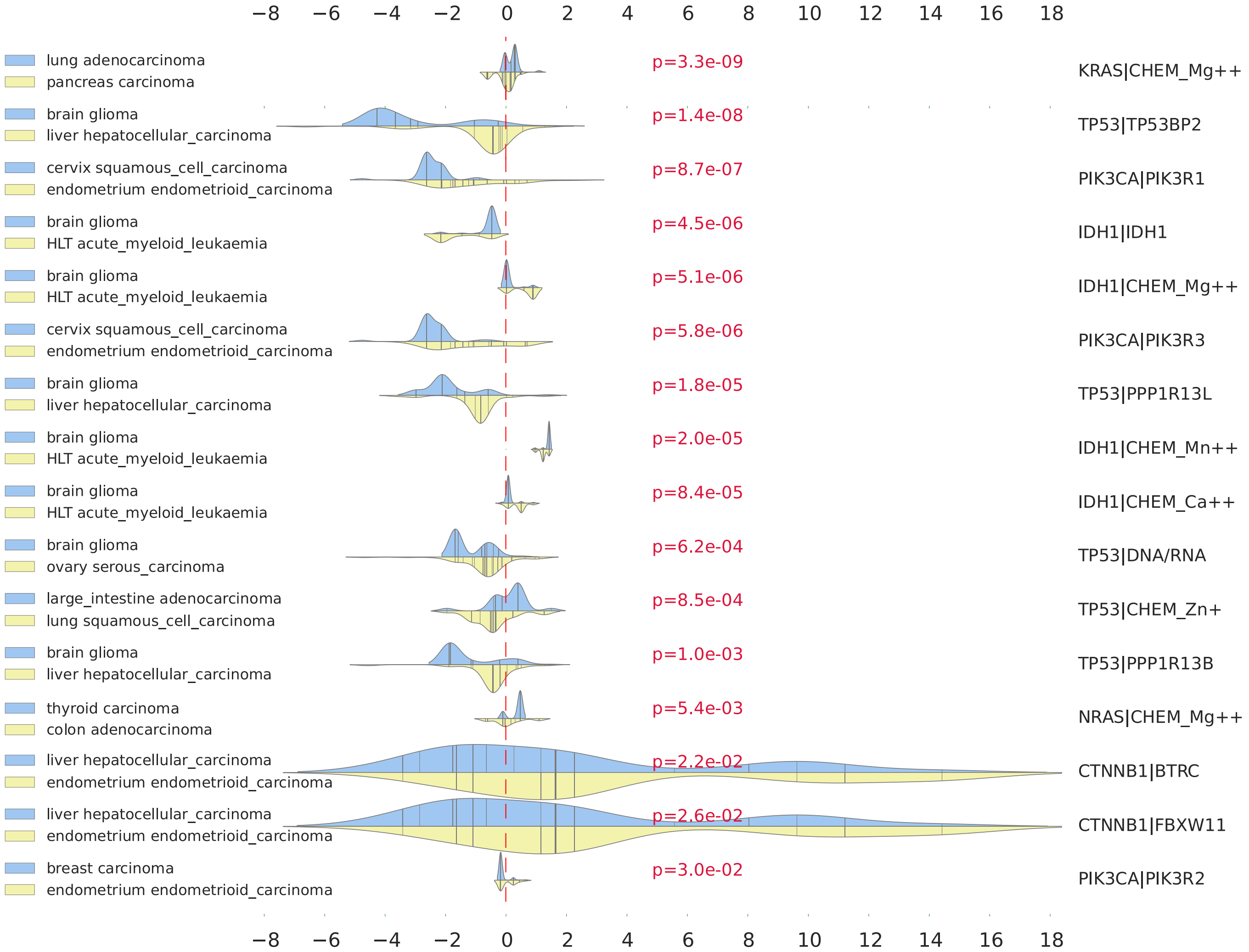
−8 −6 −4 −2 0 2 4 6 8 10 12 14 16 18
lung adenocarcinoma
pancreas carcinoma
p=3.3e-09
KRAS|CHEM_Mg++
brain glioma
liver hepatocellular_carcinoma
p=1.4e-08
TP53|TP53BP2
cervix squamous_cell_carcinoma
endometrium endometrioid_carcinoma
p=8.7e-07
PIK3CA|PIK3R1
brain glioma
HLT acute_myeloid_leukaemia
p=4.5e-06
IDH1|IDH1
brain glioma
HLT acute_myeloid_leukaemia
p=5.1e-06
IDH1|CHEM_Mg++
cervix squamous_cell_carcinoma
endometrium endometrioid_carcinoma
p=5.8e-06
PIK3CA|PIK3R3
brain glioma
liver hepatocellular_carcinoma
p=1.8e-05
TP53|PPP1R13L
brain glioma
HLT acute_myeloid_leukaemia
p=2.0e-05
IDH1|CHEM_Mn++
brain glioma
HLT acute_myeloid_leukaemia
p=8.4e-05
IDH1|CHEM_Ca++
brain glioma
ovary serous_carcinoma
p=6.2e-04
TP53|DNA/RNA
large_intestine adenocarcinoma
lung squamous_cell_carcinoma
p=8.5e-04
TP53|CHEM_Zn+
brain glioma
liver hepatocellular_carcinoma
p=1.0e-03
TP53|PPP1R13B
thyroid carcinoma
colon adenocarcinoma
p=5.4e-03
NRAS|CHEM_Mg++
liver hepatocellular_carcinoma
endometrium endometrioid_carcinoma
p=2.2e-02
CTNNB1|BTRC
liver hepatocellular_carcinoma
endometrium endometrioid_carcinoma
p=2.6e-02
CTNNB1|FBXW11
breast carcinoma
endometrium endometrioid_carcinoma
p=3.0e-02
PIK3CA|PIK3R2
−8 −6 −4 −2 0 2 4 6 8 10 12 14 16 18

Figure S4

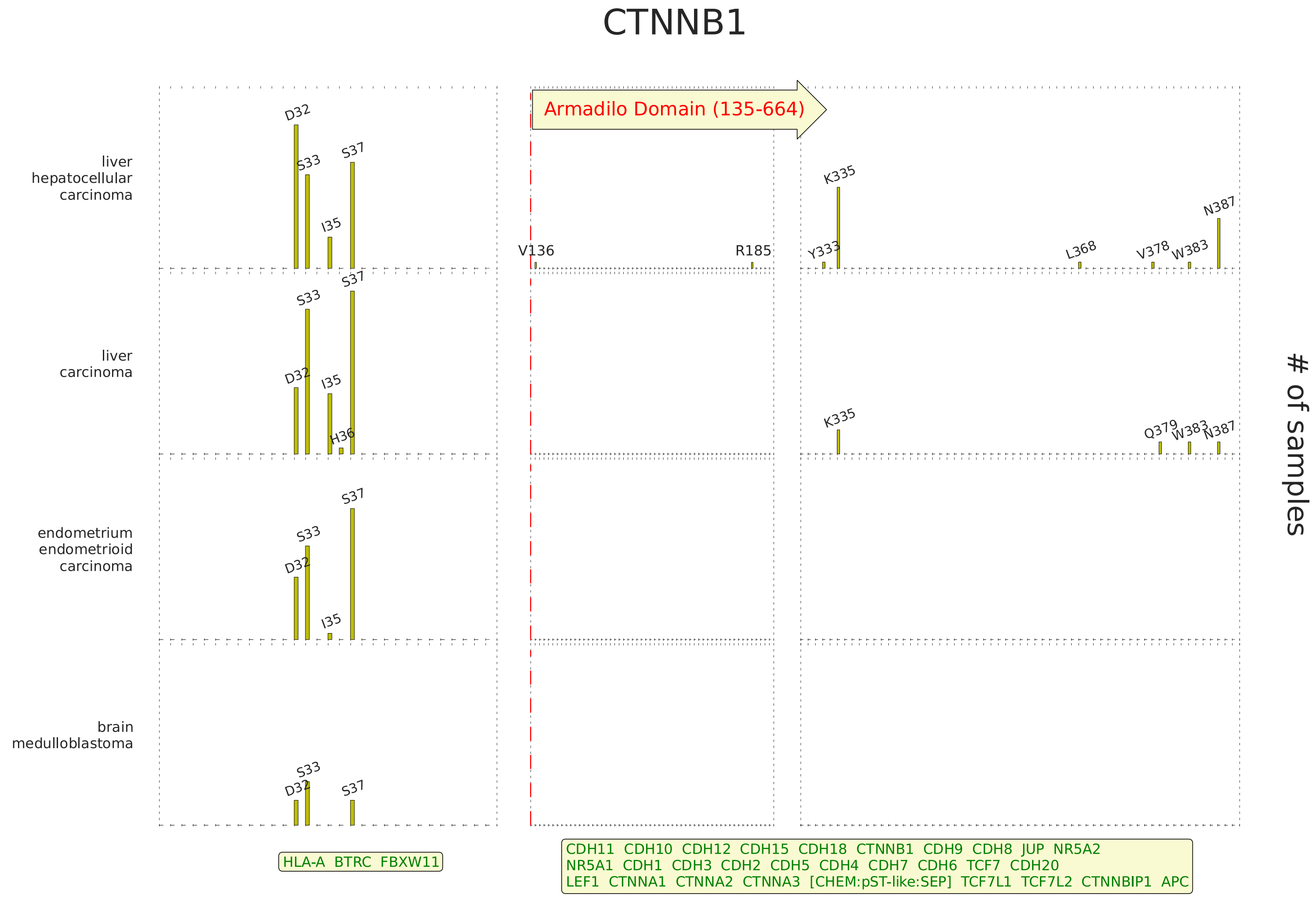
CTNNB1
Armadilo Domain (135-664)
liver hepatocellular carcinoma
liver carcinoma
endometrium endometrioid carcinoma
brain medulloblastoma
# of samples
D32
S33
I35
H36
S37
V136
R185
Y333
K335
L368
V378
Q379
W383
N387
HLA-A BTRC FBXW11
CDH11 CDH10 CDH12 CDH15 CDH18 CTNNB1 CDH9 CDH8 JUP NR5A2
NR5A1 CDH1 CDH3 CDH2 CDH5 CDH4 CDH7 CDH6 TCF7 CDH20
LEF1 CTNNA1 CTNNA2 CTNNA3 [CHEM:pST-like:SEP] TCF7L1 TCF7L2 CTNNBIP1 APC

Figure S5

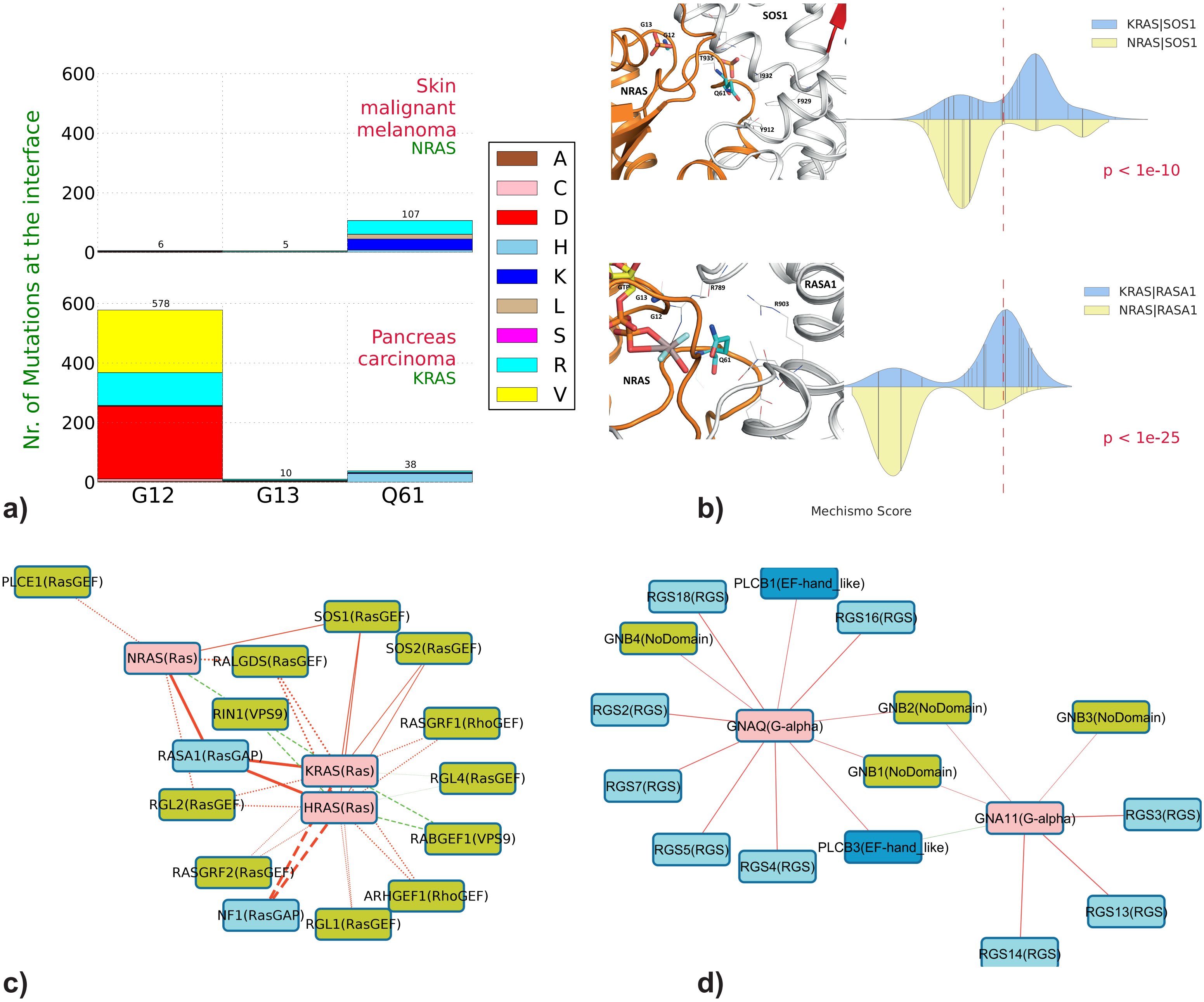

Nr. of Mutations at the interface
600
400
200
0
Skin malignant melanoma
NRAS
6
5
107
578
10
38
Pancreas carcinoma
KRAS
G12
G13
Q61
A
C
D
H
K
L
S
R
V
a)
SOS1
NRAS
KRAS|SOS1
NRAS|SOS1
p < 1e-10
RASA1
NRAS
KRAS|RASA1
NRAS|RASA1
p < 1e-25
b)
Mechismo Score
PLCE1(RasGEF)
SOS1(RasGEF)
NRAS(Ras)
RALGDS(RasGEF)
SOS2(RasGEF)
RIN1(VPS9)
RASGRF1(RhoGEF)
RASA1(RasGAP)
KRAS(Ras)
RGL4(RasGEF)
RGL2(RasGEF)
HRAS(Ras)
RABGEF1(VPS9)
RASGRF2(RasGEF)
ARHGEF1(RhoGEF)
NF1(RasGAP)
RGL1(RasGEF)
c)
RGS18(RGS)
PLCB1(EF-hand_like)
RGS16(RGS)
GNB4(NoDomain)
RGS2(RGS)
GNAQ(G-alpha)
GNB2(NoDomain)
GNB3(NoDomain)
GNB1(NoDomain)
RGS7(RGS)
GNA11(G-alpha)
RGS3(RGS)
RGS5(RGS)
RGS4(RGS)
PLCB3(EF-hand_like)
RGS13(RGS)
RGS14(RGS)
d)

Figure S6

Pan cancer

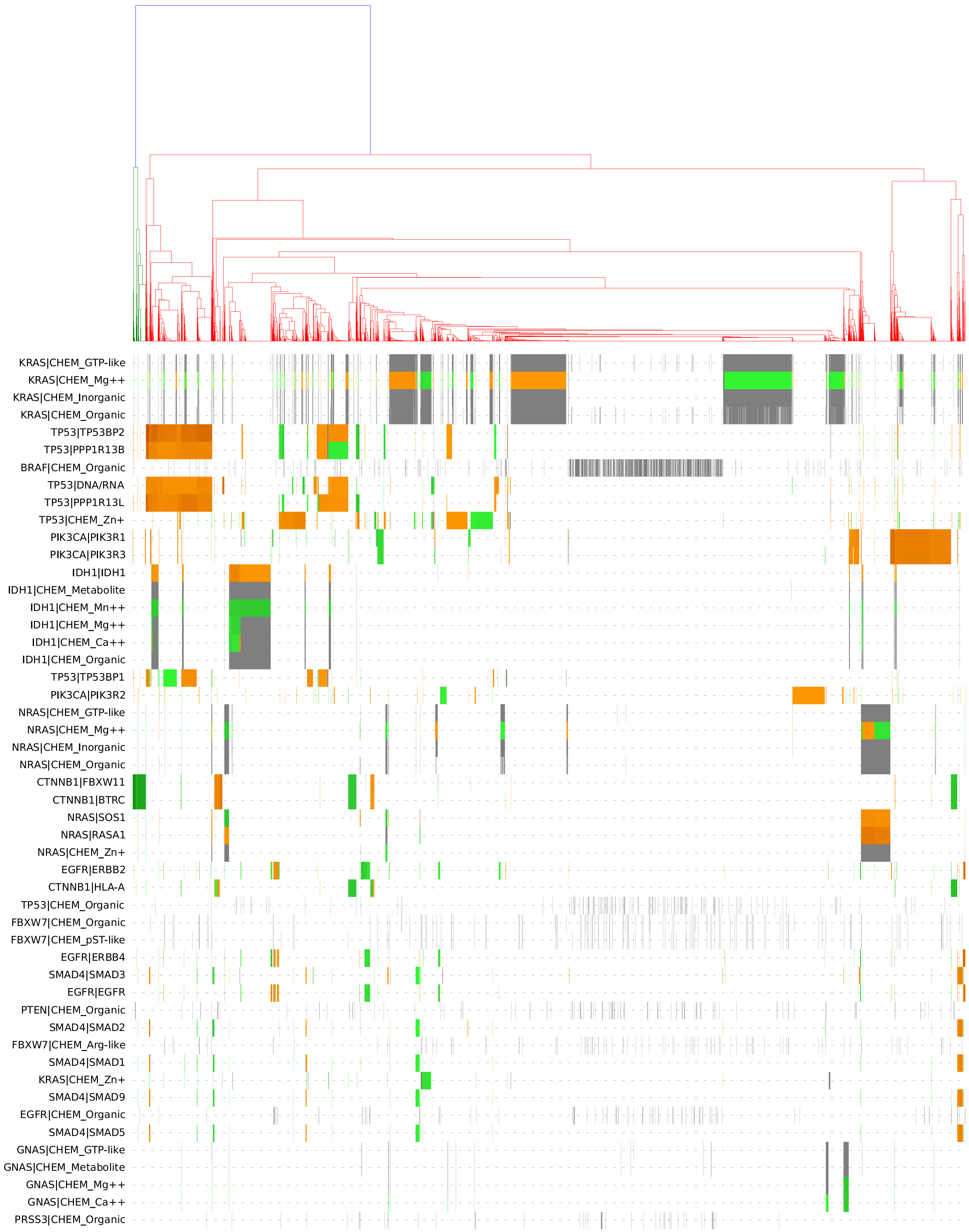

Figure S7

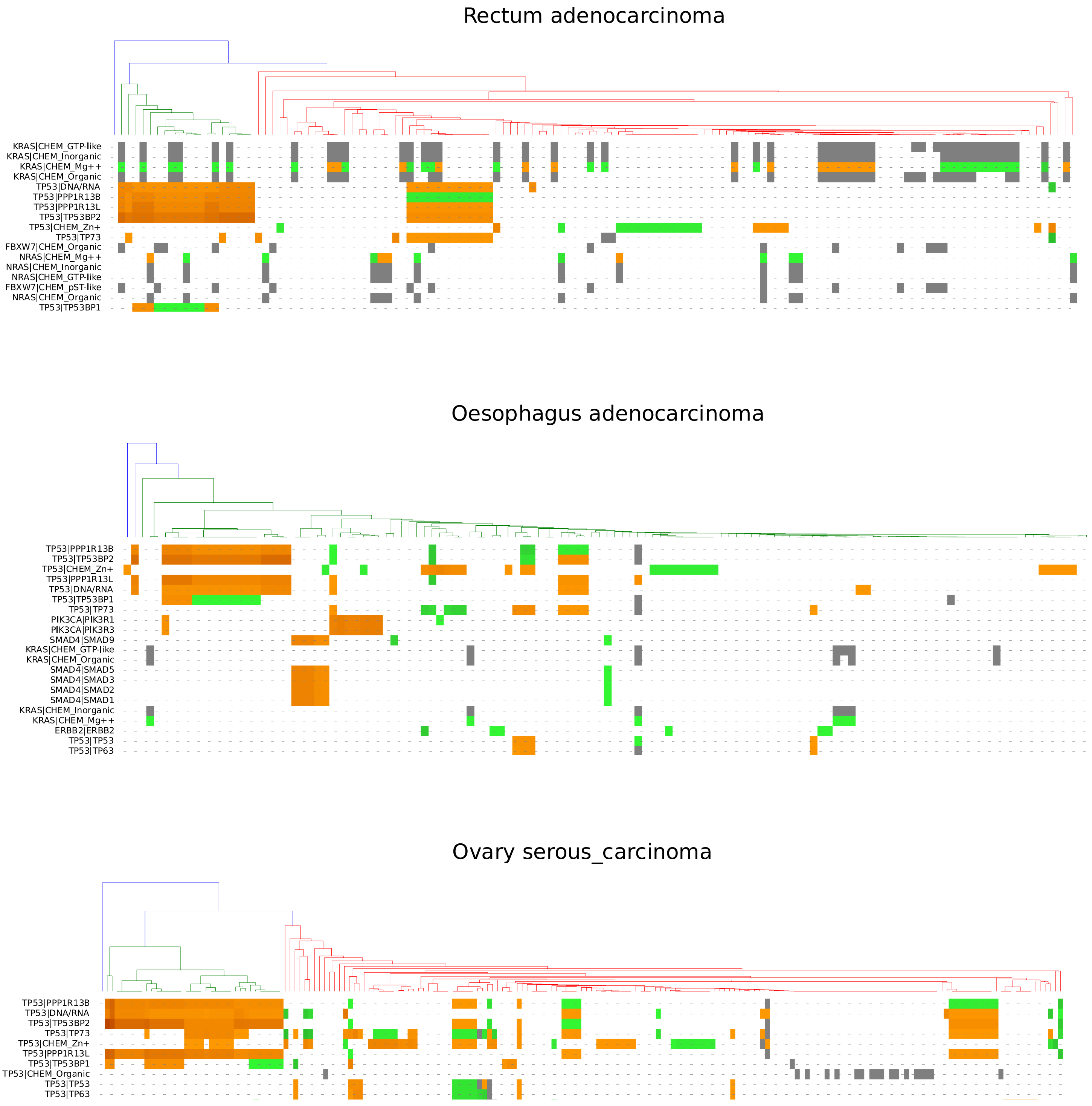

Rectum adenocarcinoma
KRAS|CHEM_GTP-like
KRAS|CHEM_Inorganic
KRAS|CHEM_Mg++
KRAS|CHEM_Organic
TP53|DNA/RNA
TP53|PPP1R13B
TP53|PPP1R13L
TP53|TP53BP2
TP53|CHEM_Zn+
TP53|TP73
FBXW7|CHEM_Organic
NRAS|CHEM_Mg++
NRAS|CHEM_Inorganic
NRAS|CHEM_GTP-like
FBXW7|CHEM_pST-like
NRAS|CHEM_Organic
TP53|TP53BP1
Oesophagus adenocarcinoma
TP53|PPP1R13B
TP53|TP53BP2
TP53|CHEM_Zn+
TP53|PPP1R13L
TP53|DNA/RNA
TP53|TP53BP1
TP53|TP73
PIK3CA|PIK3R1
PIK3CA|PIK3R3
SMAD4|SMAD9
KRAS|CHEM_GTP-like
KRAS|CHEM_Organic
SMAD4|SMAD5
SMAD4|SMAD3
SMAD4|SMAD2
SMAD4|SMAD1
KRAS|CHEM_Inorganic
KRAS|CHEM_Mg++
ERBB2|ERBB2
TP53|TP53
TP53|TP63
Ovary serous_carcinoma
TP53|PPP1R13B
TP53|DNA/RNA
TP53|TP53BP2
TP53|TP73
TP53|CHEM_Zn+
TP53|PPP1R13L
TP53|TP53BP1
TP53|CHEM_Organic
TP53|TP53
TP53|TP63

Figure S8

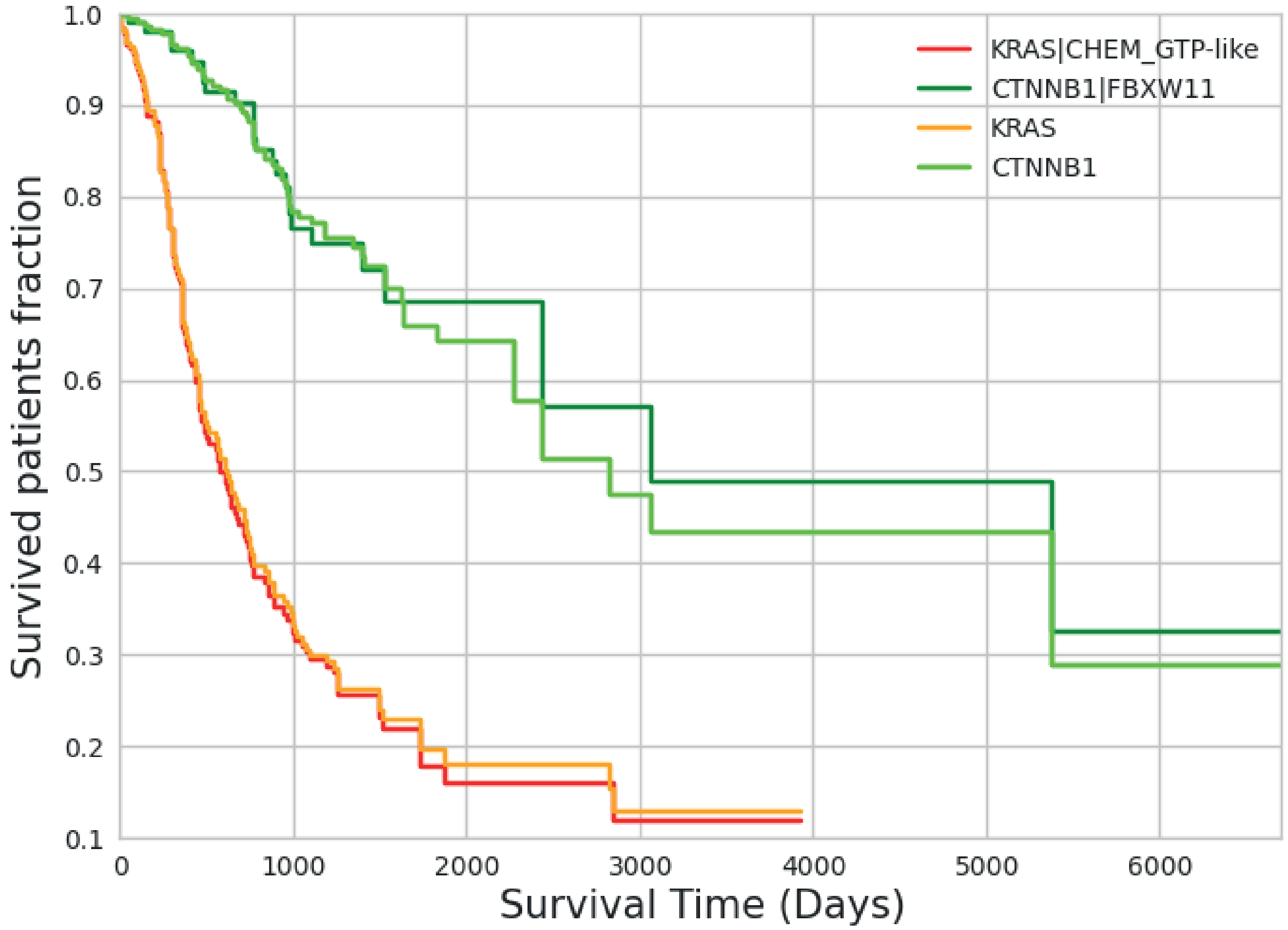

KRAS|CHEM_GTP-like
CTNNB1|FBXW11
KRAS
CTNNB1
Survived patients fraction
1.0
0.9
0.8
0.7
0.6
0.5
0.4
0.3
0.2
0.1
0
1000
2000
3000
4000
5000
6000
Survival Time (Days)

Figure S9
